# Relation between therapeutic response and side effects induced by methylphenidate as observed by parents and teachers of children with ADHD

**DOI:** 10.1186/1471-244X-11-70

**Published:** 2011-04-21

**Authors:** James Lee, Natalie Grizenko, Venkataramana Bhat, Sarojini Sengupta, Anna Polotskaia, Ridha Joober

**Affiliations:** 1 Department of Psychiatry, McGill University, Montreal, Canada; 2 Department of Human Genetics, McGill University, Montreal, Canada; 3 Department of Neurology and Neurosurgery, McGill University, Montreal, Canada; 4 Department of Child Psychiatry, Douglas Mental Health University Institute, Montreal, Canada

## Abstract

**Background:**

The desired (therapeutic) and undesired (side) effects of methylphenidate might have underlying correlations. The aim of this study was to explore the strength and the possible sources of these correlations.

**Methods:**

One hundred and fifty-seven children with ADHD (6-12 years) were administered placebo and methylphenidate (0.5 mg/kg in a divided b.i.d. dose), each for a one-week period, in a double-blind, crossover trial. Therapeutic response was assessed using the Conners' Global Index for parents (CGI-Parents) and teachers (CGI-Teachers), while side effects were assessed using the Barkley Side Effects Rating Scale (SERS).

**Results:**

The side effect profile as assessed by the SERS was similar to that of previous studies with insomnia, decreased appetite, and headaches showing significant treatment effects (p < 0.005). These "somatic/physical" side effects did not correlate with CGI-Parents or CGI-Teachers. However, the side effects of "irritability", "proneness to crying", and "anxiousness" showed significant relationships with CGI-Parents. These "mood/anxiety" side effects showed no significant correlations with the CGI-Teachers.

**Conclusion:**

The greater "mood/anxiety" side effects on methylphenidate and placebo, the less the parents observe improvement of their children while treated with methylphenidate. This suggests that the correlations between "mood/anxiety" side effects and poor response to treatment may be driven by observer effects rather than biological commonalities between therapeutic and side effects of methylphenidate.

## Background

Attention-deficit/hyperactivity disorder (ADHD) is a common neurobehavioural disorder, characterized by inattention, and/or impulsivity/hyperactivity, and emotional instability. It is one of the most prevalent childhood psychiatric disorders, affecting 5-6% of school-aged children worldwide[1].

The reported diagnosis of the disorder has increased several-fold in the past 2 decades, and there has been lingering public concern over the concurrent increase in prescriptions for stimulant medication for children who are afflicted by the disorder[2-4]. The most common medication prescribed for children with ADHD is the psychostimulant methylphenidate (MPH)[5-8]. MPH has been shown to exhibit its therapeutic response in approximately 70% of patients [9-11], though it also has a number of known side effects[12,13]. Therapeutic response to MPH includes a reduction of motor hyperactivity, more focused task-oriented behavior, reduction of impulsive behavior and emotional lability[14-16]. It has also been reported that MPH may improve neuropsychological performance on measures of executive function[17]. The extent of therapeutic response to MPH may vary from one patient to the other.

Although therapeutic response to MPH has been widely investigated, there have been only a few studies examining the side effect profile for MPH. These studies have noted a side effect profile characterized by a significant increase in frequency and severity of insomnia, decreased appetite, headaches, and stomachaches, when compared to placebo [12,18-20]. These effects are also variable from one patient to the other, and the correlations of these side effects with therapeutic effects were not explored in previous studies.

MPH has been shown to block the dopamine (DA) and norepinephrine (NE) transporters[21], resulting in increased synaptic DA and NE concentration in different brain regions including the prefrontal cortex[22]. This increased neurotransmitter concentration likely results in the modulation of various behaviors, both desired (therapeutic) and undesired (side effects) and physical manifestations. Thus, it may be hypothesized that therapeutic and undesirable effects of MPH may be correlated through common biological mechanisms. In addition, the evaluation of the therapeutic response and side effects is often reported by the parents of the child with ADHD. This may also lead to some correlation between therapeutic and side effects.

The aim of this study was to explore the correlation between the side effects observed while the child was treated with MPH and the therapeutic response as judged by parents. If such a correlation exists, it may reflect both common biological mechanisms underlying therapeutic and side effects as well as correlation in parental assessment of therapeutic and side effects. We also postulated that if the correlation between therapeutic and side effects is at least partly due to observer (parents) effects, it will be stronger for the correlation between parent's evaluation of therapeutic and side effects, compared to the correlation between therapeutic effects as assessed by teachers and side effects as assessed by parents.

## Methods

### Subjects

Children between the ages of 6 and 12 years of age were recruited from the child psychiatry outpatient clinic and the Disruptive Behavior Disorders Program at the Douglas University Institute of Mental Health, an affiliate psychiatric teaching Institute of McGill University in Montreal. The children were referred from schools, specialized care facilities, general practitioners or pediatricians. These children and their parents were interviewed by an experienced child psychiatrist. Approximately 95% of all eligible patients who met the study criteria agreed to participate with relevant data obtained from 157 patients. A wide spectrum of severity was observed from mild to the severely ill.

### Screening

All children were diagnosed with ADHD, in concordance with *DSM-IV *criteria[23]. The diagnosis was based on the Diagnostic Interview Schedule for Children Version 4 (DISC-IV) [24](parent report), and a clinical interview by a child psychiatrist. Exclusion criteria for the study included an IQ less than 70 on the WISC-III[25], Tourette's syndrome (TS), pervasive developmental disorder, and psychosis. Children were also excluded from the study if they were concurrently prescribed any medication other than MPH or had a previous history of intolerance or allergic reactions to psychostimulants. None of the children had to be excluded from the study due to severity of side effects experienced from any previous treatment. Written informed consent was provided by the parents, and all children verbally agreed to participate in the trial with MPH. Approval for the study was granted by the Research Ethics Board of Douglas Hospital.

### Assessments

Parents completed a Child Behavioral Checklist[26], which assesses several behavioral domains, and the Conners' Global Index for parents (CGI-Parents)[27]. The CGI-Parents is a widely used rating scale to assess symptoms of ADHD and other psychopathology in children between 3 and 17 years of age. The CGI scale is comprised of 10 items representing the Hyperactivity Index of the original Conners' scale. Each of the items describes a behavior that is rated on a 4-point Likert scale from 0 (not at all true) to 3 (very much true). The CGI-Parents is comprised of 2 factors: 'Emotional lability' and 'Restless-impulsive behavior'. Raw total and factor scores are transformed into normalized T-scores. A score of 65 or higher is considered to be clinically significant. The teachers also completed the CGI-teacher[28], which is equivalent to CGI-parents and has the same metric characteristics.

Parents also completed the Side Effects Rating Scale[12], which is composed of 17 side effects commonly associated with MPH treatment. Side effects were ranked on a 9-point scale from mild (score = 1) to most severe (score = 9). Scores above 7 were considered to be severe.

### Design

The study was a double-blind, placebo-controlled, crossover, randomized trial. Baseline assessments were made following a two-week washout period and 1 week before the trial began, while the child was not on any medication. Children then received, by random assignment, either placebo or 0.5 mg/kg/day MPH in a divided b.i.d. dose over a 1-week period, after which they were crossed over for the second week treatment. All treatments were prepared in identical gelatin capsules by a pharmacist who was not otherwise involved in the research project. Treatments (MPH and placebo) were packaged in individual blisters, which were clearly labeled and given to parents on the first day of the study. Blister packs were collected at the end of the two-week study to verify the compliance to treatment.

### Statistical Analysis

Chi Squared analyses were conducted to contrast frequencies of the 17 side effects, as well as the frequencies of severe side effects, between the placebo and active conditions. Repeated measures analysis of variance (ANOVA) was used to detect the effects of treatment, order of treatment, and the interaction between these two variables on the mean severity ratings for each of the 17 side effects. Effect size (Cohen's d) was also determined from the mean severity ratings and standard deviation of the side effects observed in the placebo and active conditions. Pearson correlations were conducted to investigate correlations between the side effect scores and the response to the treatment, as reported by parents and teachers, for each of the different side effects. For each statistical analyses, significance was judged at α = 0.005, to account for multiple testing (0.05/17 comparisons ~ 0.005).

## Results

### Demographic and clinical characteristics of sample

As expected, children treated with MPH as compared to placebo had significant improvement in ADHD symptoms as assessed by parents and teachers (p < 0.005). Among the 157 parent evaluations, the majority of the evaluations were done by mothers (n = 137). Only a minority of children were evaluated by fathers alone (n = 8) or both parents (n = 12). Table 1 shows the demographic and clinical characteristics of the patients. 46.7%, 42.4%, 10.9% of children were diagnosed with the combined, inattentive and hyperactive types of ADHD respectively. The frequency of oppositional defiant disorder and conduct disorder were 41.8% and 24.2%, respectively.

**Table 1:** Clinical and demographic characteristics of children with attention deficit hyperactivity disorder

Clinical characterisitics	Mean (SD)
Age, y	8.97 (1.82)

IQ (WISC-III Full Scale)	99.88 (14.94)

Mother's age, y	36.36 (6.18)

Mother's education, y	12.57 (3.16)

CBCL scales	

Aggression	72.76 (11.94)

Attention Problems	71.70 (9.62)

CPRS scales	

Restless-Impulsive	75.35 (9.62)

Emotional Lability	68.03 (12.83)

CTRS scales	

Restless-Impulsive	68.45 (10.91)

Emotional Lability	67.55 (16.13)

*WISC-III, Wechsler Intelligence Scale for Children; CBCL, Child Behavior Checklist; CPRS, Conners Parent Rating Scale; CTRS, Conners Teacher Ratings Scale.

The percent occurrence for all 17 side effects listed in the Side Effect Rating Scale (SERS) was calculated for those who had parent ratings of 1 or higher. The percent occurrence was also calculated for parent ratings of 7 or higher to establish the frequency of severe side effects to the treatments. The results are shown in Table 2. Of the 17 side effects, only *insomnia *and *decreased appetite *were significantly more frequent (p < 0.005), in the week of treatment with MPH compared to placebo. However, the percentage of children experiencing *headaches *and the *talks less *side effects increased by 11% and 10%, respectively (p < 0.05). Interestingly, the side effect of *euphoria *decreased in incidence from 31% to 20% (p < 0.05). The remaining 12 side effects did not show a significant change in frequency over the placebo and medication conditions. Among the severe side effects, significant difference between placebo and MPH weeks were observed for *decreased appetite *(4% *versus *17%, p < 0.005) and *severe headaches *(2% *versus *8%, p < 0.05).

**Table 2:** Percentage of subjects displaying each of the 17 side effects of methylphenidate during each treatment condition (Parent Ratings)*

Side Effect	Total Side Effects			Severe Side Effects		
	**Placebo**	**Active**	**P-value**	**Placebo**	**Active**	**P-value**

Insomnia (%)	25	47	0,00	7	13	0,08

Nightmares (%)	15	12	0,39	2	1	0,68

Stares a lot (%)	26	31	0,30	4	4	0,96

Talks less (%)	13	23	0,01	2	4	0,49

Uninterested (%)	15	14	0,69	2	3	0,46

Decreased Appetite (%)	20	49	0,00	4	17	0,00

Irritable (%)	52	54	0,68	12	10	0,57

Stomachaches (%)	17	25	0,10	4	4	0,92

Headaches (%)	19	31	0,01	2	8	0,02

Drowsiness (%)	8	14	0,09	0	2	0,08

Sadness (%)	27	30	0,57	4	8	0,09

Prone to crying (%)	32	43	0,05	7	6	0,88

Anxious (%)	37	34	0,54	7	4	0,25

Bites fingernails (%)	21	22	0,86	8	8	0,76

Euphoria (%)	31	20	0,03	8	5	0,21

Dizziness (%)	7	5	0,39	0	1	0,15

Tics/Nervous movements (%)	14	15	0,93	4	2	0,33

* % refers to the percentage of subjects in whom the side effect was rated 1 or higher on the scale of severity (1 to 9); % severe refers to the percentage of subjects in whom the side effect was rated 7 or higher.

The mean severity ratings by parents for each of the 17 side effects were subjected to 2-way repeated measures ANOVA to analyze the effect of treatment and to test for any effect of order of treatment. The results are shown in Table 3. It was observed that the side effects that significantly increased in severity with MPH treatment were *decreased appetite*, *insomnia*, and *headaches *(in decreasing order of effect size), at p < 0.005. The side effects of *talks less *and euphoria also had significant changes, at p < 0.05, with the former increasing nearly two-fold from 0.50 to 0.98, and the latter decreasing from 1.49 to 0.98. The effect of the order of treatment, and its the interaction with the treatment effect were not statistically significant.

**Table 3:** Mean Severity Ratings by Parents for Each of 17 Side Effects for Each Drug Condition.

Side Effect	Placebo mean ± (SD)	MPH mean ± (SD)	Treatment Effect	P-value	Order Effect	P-value	Treatment and Order Interaction	P-value	Effect Size
Decreased appetite	0,84 ± 1,95	2,55 ± 3,06	F = 59,9	0,00	F = 0,1	0,71	F = 2,3	0,13	0,67

Insomnia	1,18 ± 2,35	2,25 ± 2,94	F = 19,7	0,00	F = 1,3	0,25	F = 0,1	0,73	0,40

Headaches	0,60 ± 1,59	1,28 ± 2,45	F = 9,6	0,002	F = 0,8	0,38	F = 0,4	0,51	0,33

Talks less	0,50 ± 1,61	0,98 ± 2,04	F = 5,2	0,02	F = 3,5	0,06	F = 0,4	0,53	0,26

Drowsiness	0,33 ± 1,21	0,52 ± 1,47	F = 2,5	0,11	F = 0,7	0,41	F = 0,2	0,67	0,14

Sadness	1,03 ± 2,00	1,27 ± 2,40	F = 1,1	0,30	F = 0,6	0,43	F = 2,6	0,11	0,11

Anxious	1,55 ± 2,50	1,31 ± 2,19	F = 1,3	0,26	F = 0,3	0,59	F = 5,0	0,03	0,10

Prone to crying	1,36 ± 2,41	1,61 ± 2,40	F = 0,9	0,35	F = 0,5	0,48	F = 4,6	0,03	0,10

Nightmares	0,43 ± 1,38	0,31 ± 1,19	F = 0,6	0,46	F = 2,0	0,16	F = 1,2	0,27	0,09

Stomachaches	0,67 ± 1,86	0,83 ± 1,90	F = 0,5	0,48	F = 6,7	0,01	F = 1,1	0,30	0,09

Stares a lot	1,05 ± 2,04	1,17 ± 2,09	F = 0,4	0,54	F = 0,3	0,61	F = 0,01	0,91	0,06

Uninterested	0,55 ± 1,50	0,65 ± 1,86	F = 1,0	0,33	F = 0,2	0,62	F = 0,8	0,37	0,06

Bites fingernails	1,15 ± 2,50	1,11 ± 2,40	F = 0,03	0,85	F = 0,6	0,45	F = 1,1	0,29	0,02

Dizziness	0,19 ± 0,82	0,20 ± 1,00	F = 0,0	0,98	F = 1,0	0,32	F = 0,8	0,36	0,01

Irritable	2,42 ± 2,85	2,41 ± 2,77	F = 0,2	0,64	F = 0,2	0,69	F = 2,1	0,15	0,00

Tics/nervous Movements	0,64 ± 1,77	0,60 ± 1,63	F = 0,1	0,75	F = 1,3	0,26	F = 0,03	0,85	0,02

Euphoria	1,49 ± 2,55	0,98 ± 2,17	F = 5,6	0,02	F = 0,01	0,92	F = 0,1	0,71	0,21

Side Effects are Ordered by Effect Size.

Effect Size (Cohen's d) value from the means and standard deviations of Placebo and MPH.

Pearson correlations were conducted between side effects assessed by parents and the CGI-P ratings (as well as the factors scores for Restless-Impulsive and Emotional Lability), for each of the placebo and MPH weeks (Table 4). The CGI-P response to MPH was calculated as the difference between CGI-P during the placebo week and during the MPH week. Significant negative correlations were observed between the CGI-P response to MPH and the SERS parent ratings (placebo - MPH) for *irritability, prone to crying*, and *anxiousness *(p < 0.005). The poorer the therapeutic response, the higher these side effects were. Interestingly, side effects that revealed significant results for frequency and mean severity ratings over placebo (*insomnia, decreased appetite, headaches*) did not show any significant correlations with the parent ratings for the response to treatment.

**Table 4:** Correlation Between Therapeutic Response (CGI-P*) and Side Effects as Rated by Parents

	Placebo				Active				Placebo-Active			
**Side Effect**	**CGI-P (RI)**	**P-value**	**CGI-P (EL)**	**P-value**	**CGI-P (RI)**	**P-value**	**CGI-P (EL)**	**P-value**	**CGI-P (RI)**	**P-value**	**CGI-P (EL)**	**P-value**

Insomnia	-0,13	0,10	-0,13	0,11	0,03	0,75	0,06	0,43	0,04	0,67	0,16	0,06

Nightmares	-0,14	0,08	-0,13	0,12	-0,02	0,78	-0,15	0,07	0,03	0,75	-0,10	0,25

Stares a lot	-0,07	0,37	0,06	0,44	-0,05	0,53	-0,03	0,74	-0,09	0,30	0,00	0,99

Talks less	0,01	0,88	0,06	0,47	0,07	0,41	0,10	0,20	0,12	0,15	0,10	0,23

Uninterested	-0,11	0,17	-0,09	0,27	-0,02	0,84	-0,01	0,94	-0,08	0,34	-0,03	0,74

Decreased Appetite	0,07	0,41	-0,10	0,21	0,14	0,10	-0,01	0,87	0,17	0,04	0,02	0,83

Irritable	-0,18	0,03	-0,19	0,02	-0,19	0,02	-0,15	0,08	-0,24	0,00	-0,26	0,00**

Stomachaches	0,02	0,79	-0,05	0,58	0,13	0,12	0,02	0,77	0,19	0,03	0,06	0,47

Headaches	-0,03	0,71	0,00	0,96	-0,16	0,05	-0,14	0,10	-0,13	0,11	-0,17	0,05

Drowsiness	-0,17	0,04	-0,08	0,31	0,14	0,10	0,00	0,97	0,07	0,43	-0,09	0,28

Sadness	-0,15	0,07	-0,20	0,01	0,03	0,68	-0,13	0,11	-0,04	0,65	-0,21	0,01

Prone to crying	-0,09	0,28	-0,18	0,03	-0,03	0,70	-0,16	0,04	-0,07	0,42	-0,39	0,00**

Anxious	-0,20	0,02	-0,08	0,35	-0,01	0,90	-0,12	0,16	-0,20	0,02	-0,30	0,00**

Bites fingernails	-0,08	0,31	0,00	0,96	-0,02	0,82	0,09	0,27	-0,01	0,93	0,08	0,37

Euphoria	-0,07	0,37	-0,06	0,43	0,00	1,00	0,12	0,14	-0,11	0,22	0,00	0,99

Dizziness	-0,01	0,89	0,03	0,72	0,00	0,97	0,02	0,78	0,01	0,95	0,03	0,76

Tics/Nervous movements	-0,02	0,84	-0,02	0,78	-0,04	0,59	-0,21	0,01	-0,10	0,25	-0,18	0,04

* CGI-P, Conners Global Index-Parents; RI, Restless-Impulsive; EL, Emotional Lability, ** p < 0,005

Table 5 shows Pearson correlations between side effects assessed by parents during the placebo week and the MPH week and CGI-T ratings during the placebo week and the MPH week. Only one significant negative correlation was found between the CGI-T (restless-impulsive factor) to MPH and the SERS parent ratings (placebo) for tics/nervous movements (p < 0.005).

**Table 5:** Correlation Between Therapeutic Response (CGI-T*) and Side Effects as Rated by Parents

	Placebo				Active				Placebo-Active			
**Side Effect**	**CGI-P (RI)**	**P-value**	**CGI-P (EL)**	**P-value**	**CGI-P (RI)**	**P-value**	**CGI-P (EL)**	**P-value**	**CGI-P (RI)**	**P-value**	**CGI-P (EL)**	**P-value**

Insomnia	0,19	0,03	0,08	0,39	-0,14	0,10	-0,22	0,01	0,02	0,84	-0,10	0,25

Nightmares	-0,02	0,79	0,04	0,66	0,01	0,91	0,06	0,53	-0,10	0,27	0,04	0,69

Stares a lot	-0,05	0,60	0,12	0,18	0,05	0,60	-0,09	0,32	0,02	0,85	-0,06	0,52

Talks less	-0,08	0,37	0,00	0,98	-0,07	0,44	-0,22	0,01	-0,05	0,61	-0,12	0,20

Uninterested	-0,08	0,36	-0,05	0,59	-0,03	0,74	0,00	1,00	-0,11	0,24	-0,24	0,01

Decreased Appetite	0,12	0,18	0,06	0,50	0,03	0,78	-0,05	0,61	0,12	0,18	0,03	0,74

Irritable	0,05	0,53	0,15	0,09	-0,02	0,85	0,00	0,97	-0,03	0,71	-0,14	0,11

Stomachaches	-0,02	0,84	-0,09	0,28	0,02	0,80	-0,09	0,31	-0,07	0,48	-0,13	0,14

Headaches	0,09	0,28	0,03	0,75	-0,02	0,81	-0,10	0,23	-0,01	0,93	-0,08	0,39

Drowsiness	0,08	0,36	0,04	0,62	0,05	0,58	-0,05	0,61	0,11	0,24	0,01	0,95

Sadness	0,13	0,12	0,12	0,15	0,06	0,50	0,03	0,71	0,03	0,74	-0,11	0,21

Prone to crying	-0,14	0,10	-0,02	0,82	0,01	0,92	-0,07	0,43	-0,05	0,62	-0,13	0,16

Anxious	-0,02	0,80	0,18	0,03	0,11	0,19	0,00	1,00	0,01	0,94	-0,09	0,32

Bites fingernails	-0,11	0,19	-0,04	0,69	0,12	0,16	0,01	0,91	-0,12	0,20	-0,14	0,12

Euphoria	-0,13	0,13	-0,08	0,33	0,06	0,49	0,06	0,48	-0,21	0,02	-0,15	0,09

Dizziness	0,19	0,03	0,07	0,42	-0,04	0,62	-0,09	0,29	0,06	0,48	0,05	0,62

Tics/Nervous movements	-0,30	0,00**	-0,07	0,39	0,03	0,73	0,01	0,89	-0,03	0,73	0,04	0,68

* CGI-T, Conners Global Index-Teachers; RI, Restless-Impulsive; EL, Emotional Lability, ** p < 0,005

## Discussion

The results of the present study confirmed a similar side effect profile to those of previous studies [12,13,29], with the side effects of *insomnia *and *decreased appetite *having significantly greater prevalence in the MPH condition than the placebo condition.

The purpose of the correlation analyses was to investigate any relationships between the side effects observed while on MPH and the therapeutic response, as judged by parents through the SERS and CGI-P, respectively. The analysis yielded significant negative correlations between the response to MPH (CGI-P, emotional liability) and the side effects of *irritability *(also significant correlation with CGI-P, restless-impulsive), *proneness to crying*, and *anxiousness*. However, what is striking is that the most frequently reported side effects of *insomnia*, *decreased appetite*, and *headaches *did not show a significant correlation. Thus, it appears that the more "mood/anxiety" side effects, such as irritability, proneness to crying, and anxiousness, do affect how parents judge the behavioral improvement. This interpretation of the results is further supported by the fact that side effects such as irritability, proneness to crying, and anxiousness are far more non-specific. Furthermore, these side effects did not show a significant change in frequency or severity while on MPH, when compared to placebo, despite being the most frequently reported side effects (with the exception of decreased appetite and insomnia). This could mean that it is not MPH that is causing an increase in parent-reported irritability, anxiousness, or proneness to crying, but the fact that they were given any treatment at all, whether it is in the form of MPH or placebo.

We postulated initially that the relationships found between side effects and the parent-rated therapeutic response could be due to a shared biological mechanism, meaning that with increased therapeutic effects there will be also increased side effects. However, if this were true, we will expect that the same correlations found between side effects and the parent-rated therapeutic response should have been found with the teacher-rated therapeutic response. This correspondence was not observed in the correlational analysis. Thus, the relationships between side effects and the parent-rated therapeutic response are not likely due to common biological mechanisms, but are more an effect of the parental evaluation itself. The greater "mood/anxiety" sides effects on methylphenidate and placebo, the less the parents observe improvement of their children while treated with methylphenidate.

There are several limitations to our study that should be mentioned. The first is that while the method is experimentally appropriate, giving children 0.5 mg/kg for a 1- week period deviates from common clinical practice. Studies investigating a wider dosage range and longer period of treatment would be required to determine whether stronger significant correlations between side effects induced by and therapeutic effects of methylphenidate exist.

Another limitation is the use of only the parent report for the side effects data. The American Academy of Pediatrics practice guidelines call for a multimodal assessment battery to monitor treatment effects, such as teachers, and other caretakers in the child's environment[30]. Although teachers are better informants regarding treatment response, parents are better informants regarding the adverse effects of medication[31]. This could be due to children being more comfortable complaining about adverse effects to their parents than to their teachers.

There are a few other limitations which could modify or even mediate some of the correlations observed in this study. The study design does not include a measure of parents' treatment expectations and the presence of ADHD among parents. Finally, the majority of parental evaluations were done by mothers or both parents together, and we did not have data on the teachers' gender. This precluded us from using gender (parents and teachers) as a covariate in the analyses.

## Conclusion

The conclusion of our study is of relevance to the reliability of parent reports, in that they appear to be influenced not by the more physical side effects to the MPH, but by the more non-specific behavioural side effects. These mood/anxiety side effects were just as prevalent in the placebo condition, further emphasizing the influence of the placebo effect on any medication therapy. Furthermore, the results suggested that it is not likely that there is a common biological mechanism between the therapeutic response and side effects, particularly objective/physical side effects. One possible mechanism linking mood/anxiety side effects to poor therapeutic response as judged by parents is that anxiety might be a shared trait between the child and his parents. If so, anxious parents may become more anxious when faced with giving treatment to their children, which may exacerbate the anxiety of the child during both weeks of treatment. Anxious parents tend to be hyper-vigilant about side effects in their children. If they detect that their child is more irritable, anxious or teary, they may tend to judge negatively the effect of the treatment. The results from this study emphasize the need for further examination of the many factors that may influence the evaluation of the therapeutic response to MPH and similar drugs.

## Competing interests

RJ receives consultancy honorarium from Janssen Ortho and Pfizer Canada. All other authors deny any conflict of interest with respect to this study.

## Authors' contributions

JS worked on the statistical analysis and drafted the manuscript. NG, SS and AP participated in the design of the study. VB worked on interpretation of statistical analysis and drafting of the manuscript. RJ conceived of the study, and participated in its design and coordination and helped to draft the manuscript. All authors read and approved the final manuscript.

## Pre-publication history

The pre-publication history for this paper can be accessed here:

http://www.biomedcentral.com/1471-244X/11/70/prepub

## References

[B1] Polanczyk G de Lima M Horta B Biederman J Rohde L The worldwide prevalence of ADHD: a systematic review and metaregression analysis American Journal of Psychiatry 2007 164 942 10.1176/appi.ajp.164.6.942 17541055

[B2] Remschmidt H Global consensus on ADHD/HKD Eur Child Adolesc Psychiatry 2005 14 127 137 10.1007/s00787-005-0439-x 15959658

[B3] Jensen PS Kettle L Roper MT Sloan MT Dulcan MK Are stimulants overprescribed? Treatment of ADHD in four U.S. communities J Am Acad Child Adolesc Psychiatry 1999 38 797 804 10.1097/00004583-199907000-00008 10405496

[B4] Jureidini J Does the International Consensus Statement on ADHD leave room for healthy scepticism? Eur Child Adolesc Psychiatry 2002 11 240 author reply 241-242 12557837 10.1007/s00787-002-0282-2

[B5] Kupfer DJ The Consensus Development Panel. National Institutes of Health Consensus Development Conference statement: diagnosis and treatment of Attention-Deficit/Hyperactivity Disorder (ADHD) Journal of the American Academy of Child and Adolescent Psychiatry 2000 39 182 193 10.1097/00004583-200002000-00018 10673829

[B6] Dulcan M Practice parameters for the assessment and treatment of children, adolescents, and adults with attention-deficit/hyperactivity disorder. American Academy of Child and Adolescent Psychiatry J Am Acad Child Adolesc Psychiatry 1997 36 85S 121S 9334567 10.1097/00004583-199710001-00007

[B7] Elia J Ambrosini PJ Rapoport JL Treatment of attention-deficit-hyperactivity disorder N Engl J Med 1999 340 780 788 10.1056/NEJM199903113401007 10072414

[B8] Wigal T Swanson JM Regino R Lerner MA Soliman I Stimulant medications for the treatment of ADHD: Efficacy and limitations Mental Retardation and Developmental Disabilities Research Reviews 1999 5 215 224 10.1002/(SICI)1098-2779(1999)5:3<215::AID-MRDD8>3.0.CO;2-K

[B9] Efron D Jarman F Barker M Side effects of methylphenidate and dexamphetamine in children with attention deficit hyperactivity disorder: a double-blind, crossover trial Pediatrics 1997 100 662 666 10.1542/peds.100.4.662 9310521

[B10] Greenhill LL Swanson JM Vitiello B Davies M Clevenger W Impairment and deportment responses to different methylphenidate doses in children with ADHD: the MTA titration trial J Am Acad Child Adolesc Psychiatry 2001 40 180 187 10.1097/00004583-200102000-00012 11211366

[B11] Spencer T Biederman J Wilens T Harding M O'Donnell D Pharmacotherapy of attention-deficit hyperactivity disorder across the life cycle J Am Acad Child Adolesc Psychiatry 1996 35 409 432 10.1097/00004583-199604000-00008 8919704

[B12] Barkley RA McMurray MB Edelbrock CS Robbins K Side effects of methylphenidate in children with attention deficit hyperactivity disorder: a systemic, placebo-controlled evaluation Pediatrics 1990 86 184 192 2196520

[B13] Stein MA Sarampote CS Waldman ID Robb AS Conlon C A dose-response study of OROS methylphenidate in children with attention-deficit/hyperactivity disorder Pediatrics 2003 112 e404 10.1542/peds.112.5.e404 14595084

[B14] Rapport M Denney C DuPAUL G Gardner M Attention deficit disorder and methylphenidate: normalization rates, clinical effectiveness, and response prediction in 76 children Journal of the American Academy of Child & Adolescent Psychiatry 1994 33 882 893 10.1097/00004583-199407000-00015 8083146

[B15] Tannock R Schachar R Carr R Chajczyk D Logan G Effects of methylphenidate on inhibitory control in hyperactive children Journal of Abnormal Child Psychology 1989 17 473 491 10.1007/BF00916508 2681316

[B16] Zeiner P Bryhn G Bjercke C Truyen K Strand G Response to methylphenidate in boys with attention-deficit hyperactivity disorder Acta paediatrica 1999 88 298 303 10.1111/j.1651-2227.1999.tb01100.x 10229041

[B17] Schweitzer J Lee D Hanford R Zink C Ely T Effect of methylphenidate on executive functioning in adults with attention-deficit/hyperactivity disorder: normalization of behavior but not related brain activity Biological psychiatry 2004 56 597 606 10.1016/j.biopsych.2004.07.011 15476690

[B18] Pataki CS Carlson GA Kelly KL Rapport MD Biancaniello TM Side effects of methylphenidate and desipramine alone and in combination in children J Am Acad Child Adolesc Psychiatry 1993 32 1065 1072 10.1097/00004583-199309000-00028 8407753

[B19] Fine S Johnston C Drug and placebo side effects in methylphenidate-placebo trial for attention deficit hyperactivity disorder Child Psychiatry Hum Dev 1993 24 25 30 10.1007/BF02353715 8404241

[B20] Fischer M Newby RF Assessment of Stimulant Response in ADHD Children Using a Refined Multimethod Clinical Protocol Journal of Clinical Child Psychology 1991 20 232 244 10.1207/s15374424jccp2003_2

[B21] Solanto M Neuropsychopharmacological mechanisms of stimulant drug action in attention-deficit hyperactivity disorder: a review and integration Behavioural Brain Research 94 127 152 10.1016/s0166-4328(97)00175-7 9708845

[B22] Berridge C Devilbiss D Andrzejewski M Arnsten A Kelley A Methylphenidate preferentially increases catecholamine neurotransmission within the prefrontal cortex at low doses that enhance cognitive function Biological psychiatry 2006 60 1111 1120 10.1016/j.biopsych.2006.04.022 16806100

[B23] Diagnostic and statistical manual of mental disorders 1994 Washington, DC: American Psychiatric Association

[B24] Diagnostic Interview Schedule for Children, version IV 1998 National Institute of Mental Health Accessed March 1, 2004

[B25] Wechsler D Manual for the Wechsler Intelligence Scale for Children-Third Edition 1991 San Antonio, TX: Psychological Corporation

[B26] Achenbach T Manual for the Child Behavior Checklist/4-18 and 1991 Profile 1991 University of Vermont DoP, editor. Burlington

[B27] Conners CK Sitarenios G Parker JD Epstein JN The revised Conners' Parent Rating Scale (CPRS-R): factor structure, reliability, and criterion validity J Abnorm Child Psychol 1998 26 257 268 10.1023/A:1022602400621 9700518

[B28] Conners CK Sitarenios G Parker JD Epstein JN Revision and restandardization of the Conners Teacher Rating Scale (CTRS-R): factor structure, reliability, and criterion validity J Abnorm Child Psychol 1998 26 279 291 10.1023/A:1022606501530 9700520

[B29] Barkley RA A review of stimulant drug research with hyperactive children The Journal of Child Psychology and Psychiatry 1977 18 137 165 10.1111/j.1469-7610.1977.tb00425.x 326801

[B30] Perrin J Stein M Amler R Blondis T Feldman H Clinical practice guideline: treatment of the school-aged child with attention-deficit/hyperactivity disorder Pediatrics 2001 108 1033 1044 11581465 10.1542/peds.108.4.1033

[B31] Schachar RJ Tannock R Cunningham C Corkum PV Behavioral, situational, and temporal effects of treatment of ADHD with methylphenidate J Am Acad Child Adolesc Psychiatry 1997 36 754 763 10.1097/00004583-199706000-00011 9183129

